# Recent Trends in Control Methods for Bacterial Wilt Diseases Caused by *Ralstonia solanacearum*

**DOI:** 10.1264/jsme2.ME14144

**Published:** 2015-02-26

**Authors:** Yuliar, Yanetri Asi Nion, Koki Toyota

**Affiliations:** 1 Research Center for Biology, Indonesian Institute of Sciences (LIPI)Jl. Raya Jakarta-Bogor, Km 46, Cibinong Science Center 16911Indonesia; 2 Graduate School of Bio-Applications and Systems Engineering, Tokyo University of Agriculture and Technology2–24–16 Nakacho, Koganei, Tokyo, 184–8588Japan; 3 Palangka Raya University JlYos Sudarso, Center of Kalimantan, Palangka RayaIndonesia

**Keywords:** antibiosis, biological control agent, competition, induced systemic resistance, organic amendment

## Abstract

Previous studies have described the development of control methods against bacterial wilt diseases caused by *Ralstonia solanacearum*. This review focused on recent advances in control measures, such as biological, physical, chemical, cultural, and integral measures, as well as biocontrol efficacy and suppression mechanisms. Biological control agents (BCAs) have been dominated by bacteria (90%) and fungi (10%). Avirulent strains of *R. solanacearum*, *Pseudomonas* spp., *Bacillus* spp., and *Streptomyces* spp. are well-known BCAs. New or uncommon BCAs have also been identified such as *Acinetobacter* sp., *Burkholderia* sp., and *Paenibacillus* sp. Inoculation methods for BCAs affect biocontrol efficacy, such as pouring or drenching soil, dipping of roots, and seed coatings. The amendment of different organic matter, such as plant residue, animal waste, and simple organic compounds, have frequently been reported to suppress bacterial wilt diseases. The combined application of BCAs and their substrates was shown to more effectively suppress bacterial wilt in the tomato. Suppression mechanisms are typically attributed to the antibacterial metabolites produced by BCAs or those present in natural products; however, the number of studies related to host resistance to the pathogen is increasing. Enhanced/modified soil microbial communities are also indirectly involved in disease suppression. New promising types of control measures include biological soil disinfection using substrates that release volatile compounds. This review described recent advances in different control measures. We focused on the importance of integrated pest management (IPM) for bacterial wilt diseases.

The world’s population is increasing every year. In order to meet the demands of an ever expanding human population, global crop production needs to double by 2050; however, current estimates are far below what is needed (104). Plant diseases, insects, and weeds decrease the production of crops worldwide by 36%, and diseases alone have been shown to reduce crop yields by 14% (5). Thus, the control of plant diseases contributes to increased crop production. Among plant diseases, soil-borne diseases are considered to be more limiting than seed-borne or air-borne diseases in the production of many crops and account for 10–20% of yield losses annually (120).

The top ten bacterial species have been listed based on their scientific and economic importance in plant diseases: i) *Pseudomonas syringae* pathovars, ii) *Ralstonia solanacearum*, iii) *Agrobacterium tumifaciens*, iv) *Xanthomonas oryzae* pv. *oryzae*, v) *X. campestris* pathovars, vi) *X. axonopodis* pathovars, vii) *Erwinia amylovora*, viii) *Xylella fastidiosa*, ix) *Dickeya* (former *Erwinia*) (*dadantanii* and *solani*), and x) *Pectobacterium* (former *Erwinia*) *carotovorum* (and *Pectobacterium atrosepticum*) (79).

*R. solanacearum* (Smith) Yabuuchi *et al.* (132) (syn. *Pseudomonas solanacearum* [Smith] Smith, *Burkholderia solanacearum* [Smith]) causes a vascular wilt disease and has been ranked as the second most important bacterial pathogen. It is one of the most destructive pathogens identified to date because it induces rapid and fatal wilting symptoms in host plants. The host range is extensively wide, over 200 species, and the pathogen is distributed worldwide and induces a destructive economic impact (57). Direct yield losses by *R. solanacearum* vary widely according to the host, cultivar, climate, soil type, cropping pattern, and strain. For example, yield losses vary from 0 to 91% in the tomato, 33 to 90% in the potato, 10 to 30% in tobacco, 80 to 100% in the banana, and up to 20% in the groundnut (28). Difficulties are associated with controlling this pathogen due to its abilities to grow endophytically, survive in soil, especially in the deeper layers, travel along water, and its relationship with weeds (122).

The management of bacterial wilt with physical, chemical, biological, and cultural methods has been investigated for decades. Elphinstone (28) extensively reviewed bacterial wilt in 2005, and many studies have since been conducted on this topic. We herein reviewed the same topic, but mainly based on findings published between 2005 and 2014.

Elphinstone (28) reported that over 450 studies had been published on *R. solanacearum* since the second International Bacterial Wilt Symposium was held in Guadaloupe in 1997. A broad classification on these studies showed that 24% were concerned with breeding and selection for resistance, while the remainder investigated the diversity, distribution, and host range of the pathogen (22%), disease management and control (18%), pathogenicity and host-pathogen interactions (17%), biological control (10%), detection and diagnosis of the pathogen (4%), and epidemiology and ecology (3%). Based on our reference search from books and journals between 1984 and 2014 using the Web of Science, LINK (Springer), InterScience (Wiley), SD-Science Direct (Elsevier), and Synergy (Blackwell), studies on methods regarding the biological control of bacterial wilt (54%) were the most common, followed by those on cultural practices (21%), chemical methods (8%), and physical methods (6%). Some studies also focused on integrated pest management (11%). This finding suggested that many researchers were interested in biological control.

We herein discussed the following points, (i) methods used to control bacterial wilt and their limitations, and (ii) how these methods are useful for improving crop production through the suppression of bacterial wilt.

## Methods used for crop protection

### Chemical methods (pesticides and non-pesticides)

World pesticide use exceeded 5.0 billion pounds in 2000 and 2001 (59). Herbicides account for the largest portion of the total use, followed by insecticides and fungicides. Plant disease control has been largely dependent on the use of pesticides (127). Schreinemachers *et al.* (107) reported that pesticide use per hectare, especially herbicides and fungicides/bactericides, had generally increased more than porportionally with crop output per hectare, and revealed that a 1% increase in crop output per hectare was associated with 1.8% increase in pesticide use per hectare.

Pesticides such as algicide (3-[3-indolyl] butanoic acid), fumigants (metam sodium, 1,3-dichloropropene, and chloropicrin), and plant activators generating systemic resistance on the tomato (validamycin A and validoxylamine) have been used to control bacterial wilt. The combination of methyl bromide, 1,3-dichloropropene, or metam sodium with chloropicrin significantly reduced bacterial wilt in the field from 72% to 100% and increased the yield of tobacco and the tomato. The yield of the pesticide-treated tomato was 1.7- to 2.5-fold higher than that of the untreated control (32, 105).

Edwards-Jones (27) reported that pesticides offered greater net benefits than other control methods, but this has not always been the case. For example, if farmers use pesticides carelessly or without proper knowledge, a percentage of the pesticide may remain in the environment for many years (34), become a contaminant in soil and/or groundwater (2), and be poisonous to farmers (25).

Bactericides (triazolothiadiazine [0.5 to 12 mM, in solution] (58), streptomycin sulfate [400 mg kg^−1^ of soil] (72)), other chemicals such as bleaching powders (application rate to the field, 30 kg ha^−1^) as sterilizers (108), or weak acidic electrolyzed water (40 ppm of available chlorine, in pH 5.6 solution) (137) have also been shown to effectively destroy microorganisms.

Acibenzolar-S-methyl (ASM) has been proposed to induce systemic resistance (38, 100). The combination of ASM and thymol significantly reduced the incidence of disease and increased the yield of the tomato, whereas ASM or thymol alone did not (43). Silicon (24, 65, 129) or Si and chitosan (61) reduced the incidence of bacterial wilt through induced resistance. Wang *et al.* (123) reported that Si-mediated resistance was associated with increases in the amount of microorganisms in the soil as well as soil enzyme activity (urease and acid phosphatase). The soaking of seeds in a low sodium chloride solution was previously found to increase seedling vigor and tolerance to *R. solanacearum* in the tomato (86).

The mechanism of action of non-pesticide chemicals that suppress bacterial wilt is considered to involve either induced systemic resistance or antibacterial activity. Some new methods have been reported to suppress bacterial wilt. Live microbial cells of the pathogen were captured with 10 g kg^−1^ of coated sawdust with 1% of an equimolar polymer of N-benzyl-4-vinylpyridinium chloride with styrene (PBVP-co-ST) (55) or coagulated in the soil with 10 mg kg^−1^ of a co-polymer of methyl methacrylate with N-benzyl-4-vinylpyridinium chloride at a molar ratio 3:1 (PMMA-co-BVP) (56). Infection by the bacterial wilt pathogen was prevented through bacteriostatic actions with a phosphoric acid solution (89).

Various non-pesticide chemicals have the potential to be applied in the field in order to control bacterial wilt disease because they have less damaging effects on the environment; however, economic considerations often influence the chemicals selected. Expensive chemicals and repeated applications are only possible for valuable crops that may incur substantial economic losses in the absence of treatments. Since crop yield and quality are not damaged when disease severity is low or in the absence of pathogens, a diagnosis based on economic thresholds is essential for determining whether chemical treatments are needed.

### Biological method

#### 1) Biological control agents (BCAs)

Interest in biological control has increased due to concerns over the general use of chemicals (126). The benefits of BCAs are 1) potentially self-sustaining, 2) spread on their own after initial establishment, 3) reduced input of non-renewable resources, and 4) long-term disease suppression in an environmentally friendly manner (102, 127).

The mechanisms employed by BCAs are sustained by various interactions such as competition for nutrients and space, antibiosis, parasitism, and induced systemic resistance (5, 22). Our reference survey revealed that BCAs have been dominated by bacteria (90%) and fungi (10%). Montesinos (84) found that most patented BCAs are made of bacteria. Topics regarding biocontrol agents for bacterial wilt have been separated into the following categories: isolation, screening and identification of BCAs, application methods of BCAs, improved BCAs, suppression mechanisms of BCAs, and effects of BCAs on the environment.

Previous studies showed the potential value of some promising BCAs, which are dominantly avirulent strains of *R. solanacearum* and *Pseudomonas* spp., followed by *Bacillus* spp., *Streptomyces* spp., and other species, in controlling bacterial wilt. A total of 109 strains of endophytic or rhizobacteria were recently screened for their antibacterial activities against *R. solanacearum*, and effective isolates (a total of 22) consisted of *Pseudomonas* spp. (18 isolates) and *Bacillus* sp. (2 isolates) (103). Kurabachew *et al.* (64) screened 13 out of 150 isolates of rhizobacteria based on *in vitro* antibiosis, and they were *Pseudomonas* spp., *Serratia marcescens*, and *Bacillus cereus*. Among *Bacillus* spp., the number of studies being conducted on *B. amyloliquefaciens* is increasing (21, 26, 44, 116, 124, 143). Plant growth-promoting rhizobacteria are commonly isolated from the rhizosphere of healthy plants and an interesting strategy has been reported. Huang *et al.* (45) revealed that isolates from the rhizosphere of diseased plants performed better in reducing disease incidence that those of healthy plants. In their study, the biocontrol efficacies of the antagonists were related to root colonizing capacities, but not with antibiosis *in vitro*, suggesting that root colonizing capacity may play a key role in disease suppression.

Several new or uncommon BCAs have been reported to control bacterial wilt such as *Acinetobacter* sp. (130), *Burkholderia nodosa*, *B. sacchari*, *B. tericola*, *B. pyrrocinia* (88), bacteriophages (10, 133), *Bacillus thuringiensis* (146), *Chryseobacterium daecheongense* (45), *Chryseobacterium indologenes* (42), *Chryseomonas luteola* (42), *Clostridium* sp. (82), *Delftia acidovorans* (45), *Enterobacter* sp. (130), *Flavobacterium johnsoniae* (45), *Myroides odoratimimus* (138), *Paenibacillus marcerans* (70), *P. polymyxa* (69, 74), *Pseudomonas brassicacearum* (145), *Ralstonia pickettii* (125), *Serratia* sp. (37, 131), *Sphingomonas paucimobilis* (42), *Staphylococcus auricularis* (42), *Stenotrophomonas maltophilia* (81), *Streptomyces rochei* (76), *S. virginiae* (115), and *Xenorhabdus nematophila* (52). The possible suppression mechanisms of these species are competition, induced systemic resistance, antibiosis, and the production of enzymes that degrade the cell wall and siderophores. Successful trials using BCA in the field are introduced in Table 1. Hyakumachi *et al.* (47) recently revealed that *B. thuringiensis*, a famous bioinsecticide-producing bacterium, induced defense-related genes, such as PR-1, acidic chitinase, and beta-1,3-glucanase and showed resistance against a direct inoculation with *R. solanacearum*. The expression of several salicylic acid-responsive defense-related genes was confirmed to be specifically induced (114), and also that suppression by *B. thuringiensis* may differ from the induced systemic resistance (ISR) elicited by many plant growth-promoting rhizobacteria (PGPR), in which jasmonic acid and ethylene-dependent signaling pathways mediate plant resistance to pathogens (47).

Some fungal BCAs have been reported to control bacterial wilt. In pot cultures, populations of *R. solanacearum* in the rhizosphere, on root surfaces, and in the xylem of tomato plants decreased by 26.7, 79.3, and 81.7%, respectively, following the inoculation of *Glomus versiforme*. The colonization of plants by both *R. solanacearum* and *G. versiforme* increased the contents of soluble phenols and cell-wall bound phenols in the root tissue, which may be related to ISR by the fungus (147). Another fungus, *Pythium oligandrum*, has the potential to control bacterial wilt disease, in which cell wall proteins may play an important role in the induction of resistance to *R. solanacearum*, accompanied by activation of the ethylene-dependent signaling pathway (41). Shiitake mycelia leachate was found to contain an antibiotic ingredient that suppressed the growth of *R. solanacearum in vitro* (93). In addition, three endomycorrhizal fungi (*Gigaspora margarita, Glomus mosseae, and Scutellospora* sp.) (112) and the lichen *Parmotrema tinctorum* (35) have been identified as BCAs against *R. solanacearum*.

In the inoculation methods of BCAs, pouring or drenching soil was more prevalent than other methods, whereas the biocontrol efficacy range appeared to be lower than that of the dipping of roots or seed coating method.

There are some disadvantages to BCAs. The biggest obstacle is their poor performance due to inconsistent colonization. Suppression by BCAs has been observed in a narrow range of host plants or restricted to a single pathogen or disease (127). The degree of suppression is sometimes too low to be commercially acceptable or requires uneconomically high rates of inoculums to be applied (127). Difficulties have also been associated with producing, storing, and subsequently applying BCAs. An option to overcome the storage problem is to select spore formers as BCAs (*e.g.* 21, 47, 74, 116, 124, 146).

#### 2) Organic matter

Organic amendments to soil have direct impacts on plant health and crop productivity. They are advantageous because they improve the physical, chemical, and biological properties of soil, which can have positive effects on plant growth (14).

The degradation of organic matter in soil can directly affect the viability and survival of a pathogen by restricting available nutrients and releasing natural chemical substances with varying inhibitory properties (14). Carbon released during the degradation of organic matter contributes to increasing soil microbial activity and thereby enhances the likelihood of competition effects in the soil (14). Organic amendments to soil have been shown to stimulate the activities of microorganisms that are antagonistic to pathogens (6). In addition, organic amendments often contain biologically-active molecules such as vitamins, growth regulators, and toxins, which can affect soil microorganisms. Youssef and Tartoura (139) recently reported that plant resistance against the bacteial wilt pathogen was enhanced through the augmented activities of ascorbate peroxidase, monodehydroascorbate reductase, dehydroascorbate reductase, and glutathione reductase following the application of compost.

Organic matter originates from recently living organisms and decays or is the product of decay. It is categorized into plant or animal origins, and simple organic carbons. In the previous references to an *R. solanacearum* study, different organic matter, such as plant residue (80%), animal waste (10%), and simple organic matter (10%), were shown to control bacterial wilt disease. Larkin (67) found that biological amendments were generally effective for delivering microorganisms to natural soil, resulting in a wide variety of effects on soil microbial communities depending on the particular types, numbers, and formulations of organisms added. A new approach is the suppression of bacterial wilt in an organic hydroponic system through a rhizosphere biofilm that only forms on roots in the organic system (33).

#### 2a) Plant residue controlling bacterial wilt

Several previous studies reported that bacterial wilt was suppressed by plant residues derived from, *e.g.* chili (*Capsicum annum*) (117), Chinese gall (*Rhus chinensis*) (142), clove (*Szygyum aromaticum*) (11), cole (*Brassica* sp.) (13, 90, 97), eggplant (*Solanum melongena*), (9), eucalyptus (*Eucalyptus globules*) (94, 95), geranium (*Geranium carolinianum*) (91), guava (*Psidium guajava* and *P. quineense*) (3), hinoki (*Chamaecyparis obtusa*) (141), Japanese cedar (*Cryptomeria japonica*) (46, 80), lemongrass (*Cimbopogon citratus*) (94, 95), marigold (*Tagetes patula*) (118), neem (*Azadirachta indica*) (96), palmarosa (*Cimbopogon martint*) (94, 95), pigeon pea (*Cajanus cajan*), sunn hemp (*Crotalaria juncea*) (20), tamarillo (*Cyphomandra betacea*) (92), thyme (*Thymus* spp.) (53, 99), wood wax tree (*Toxicodendron xylvestre*) (142), and worm killer (*Aristolochia bracteata*) (109). The possible mechanisms of action of the plant residues are mainly considered to be antimicrobial activities, followed by the indirect suppression of the pathogen through improved physical, chemical, and biological soil properties (20). For example, the antimicrobial compounds from *Tagetes patula* that suppressed *R. solanacearum* in an *in vitro* experiment were identified as 5-(3-buten-1-ynyl)-2,2′-bithienyl (BBT) and 5-(4-acetoxy-1-butynyl)-2,2′-bithienyl (BBTOAc) (118). Other plants such as *Cryptomeria japonica* produced sandaracopimarinol and ferruginol (80) while *Cyphomandra betacea* contained a glycosidase inhibitory protein that suppressed *R. solanacearum* in an *in vitro* experiment (92). Lansiumamide B isolated from the seeds of *Clausena lansium* suppressed tobacco bacterial wilt more than an antibiotic streptomycin when applied at a density of 100 mg kg^−1^ (71).

Previous experiments demonstrated the successful application of organic matter against bacterial wilt in greenhouses and in the field. For example, in a greenhouse experiment, when the freshly cut aerial parts of pigeon pea (*Cajanus cajan*) and crotalaria (*Crotalaria juncea*) were incorporated at concentrations of 20–30% and incubated for 30 d, they completely suppressed tomato bacterial wilt 45 d after the inoculation (20); however, the application rate of this organic matter was high and, thus, not feasible for farmers. Thymol oil derived from a thyme plant reduced bacterial wilt by 65% in the fall 2002 tomato cultivation and by 82% in fall 2003 tomato cultivation at an application rate of 0.72% in the field (53). Alfano *et al.* (8) reported that the disease suppressive effects of olive waste compost appeared to be due to the combined effects of suppression phenomena caused by the presence of microorganisms competing for both nutrients and space as well as by the activity of specific antagonistic microorganisms.

#### 2b) Animal waste controlling bacterial wilt

Although many studies have already reported that animal waste controls plant disease, few have shown that animal waste suppresses bacterial wilt disease. For example, the application of pig slurry decreased the population of *R. solanacearum* in the soil (36). The mechanisms underlying the enhanced decline of the population of this pathogen and disease suppression remains unclear; however, shifts in bacterial community profiles have been proposed. Another study suggested that the suppression of bacterial wilt by poultry and farmyard manure were related to higher microbial activity and higher numbers of cultural bacteria and fungi (50). In that study, a lower disease index was related to the poor survival of the pathogen. However, limitations are associated with the wide use of organic waste. Janvier *et al.* (51) demonstrated that the major key-points for the efficiency of organic matter in suppressing plant pathogens depended on: i) the plant-pathogen combination, ii) the rate of application, iii) the nature/type of amendment, and iv) the degree of maturity of the decomposition stage of crop residues.

#### 2c) Simple organic compounds controlling bacterial wilt

The efficacy of simple organic compounds, including amino acids, sugars, and organic acids, on bacterial wilt in the tomato was evaluated in pot experiments. The application of lysine to a pumice culture medium (0.25 mg g^−1^) and soil (2.5 mg g^−1^) reduced bacterial wilt in the tomato by 85–100% (48, 87) and by 58–100% (97), respectively. The suppression mechanism was not attributed to the induction of systemic resistance, but to shifts in the soil microbial community structure that led to the more rapid death of the pathogen (98). In contrast, riboflavin induced a series of defense responses and secondary metabolism in cell suspensions and, thus, protected tobacco against *R. solanacearum* (75). DL,-3-aminobutyric acid (BABA) also increased polyphenol oxidase activity and decreased that of catalase in tomato plants, suggesting the induction of resistance to bacterial wilt in the tomato (40). Another study showed that methyl gallate exhibited strong bactericidal effects on *R. solanacearum* (30).

### Physical methods, including biofumigation

A number of physical control methods, *e.g.* solarization and hot water treatments, have proved to be effective against *R. solanacearum*. Vinh *et al.* (121) found that soil solarization using transparent plastic mulches for 60 d prior to the planting of tomatoes reduced the incidence of bacterial wilt. Another study reported that rhizome solarization on ginger seeds for 2 to 4 h reduced bacterial wilt by 90–100% 120 d after planting, and that ginger seeds sterilized with discontinuous microwaving (10-s pulses) at 45°C reduced the incidence of wilt by 100% (63). Baptista *et al.* (16, 17) studied the mechanisms of soil solarization that reduced bacterial wilt in the tomato. Soil solarization reduced soil pH, potassium (K), sodium (Na), boron (B), and zinc contents, microbial biomass, and microbial respiration in soil, but did not significantly affect other soil chemical properties. A heat treatment at either 45°C for 2 d or a minimum temperature of 60°C for 2 h of the infected soil prior to tomato planting reduced the total bacterial population by 60–97%, that of *Ralstonia* sp. from 2 to 7×10^8^ cfu g^−1^ to 0 to 115 cfu g^−1^, and the incidence of bacterial wilt by 50–75% (62). Several parameters need to be carefully considered before the application of soil solarization can be expanded: controlling temperature or the release of volatile compounds and economical and/or practical feasibility in field.

In addition to heat treatments, cold temperatures are also sometimes effective. Bacterial wilt rarely occurs in tobacco crops planted in May or June (winter crop) in north Queensland because of cool weather conditions, whereas the disease developed when crops were planted in spring (September to November), particularly when bacterial wilt had previously occurred and crop rotation was not practiced (7). Lower moisture conditions (20–30% maximum water holding capacity) and pre-incubation at lower temperatures (4°C) reduced bacterial wilt and had a negative impact on the survival of *R. solanacearum* (50). Scherf *et al.* (106) found that *R. solanacearum* survived for 6 months in an infected geranium at a constant temperature, but declined rapidly in repeated winter temperature cycles of 2 d at 5°C followed by 2 d at −10°C. The mechanism of action responsible for the suppression of bacterial wilt by physical methods generally involves killing pathogens with high or low temperatures.

Biofumigation, which refers to the agronomic practice of using volatile chemicals released from plant residues to suppress soil-borne plant pathogens, has recently been attracting attention (61). Biofumigation is called biological soil disinfection (BSD) and the production of organic acids or heavy metal ions is involved in the suppression of pathogens (83).

Another approach is control with a high voltage electrostatic field and radio frequency electromagnetic field, in which ISR is involved in the suppression mechanism (128). Silver-coated non-woven cloth filter and a visible light source (15) or electrostatic spore precipitator ozone-saturated water (144) was developed as a sterilization device and inactivated the pathogen.

### Cultural practices

#### 1) Cultivar resistant

The growth of cultivars that are resistant to bacterial wilt is considered to be the most economical, environmentally friendly, and effective method of disease control. Breeding for resistance to bacterial wilt has been concentrated on crops of wide economic importance such as the tomato, potato, tobacco, eggplant, pepper, and peanut, and has commonly been influenced by factors such as the availability of resistance sources, their diversity, genetic linkage between resistance, and other agronomic traits, differentiation and variability in pathogenic strains, the mechanism of plant-pathogen interactions, and breeding or selection methodology (19, 28, 39). For example, the *Arabidopsis* NPR1 (non-expresser of *PR* genes) gene was introduced into a tomato cultivar, and enhanced resistance to bacterial wilt and reduced the incidence of wilt by approximately 70% 28 d after the inoculation (73). Potato genotype BP9, which is a somatic hybrid between *Solanum tuberosum* and *S. phureja*, successfully reduced bacterial wilt by 90–100% (31). Somatic hybrids between *S. melongena* cv. Dourga and two groups of *S. aethiopicum* were produced by the electrical fusion of mesophyll protoplasts and were found to be tolerant to *R. solanacearum*. Public acceptance in Japan is needed prior to the commercial use of such genetically modified crops.

Prior *et al.* (101) showed that resistant plants were heavily invaded by *R. solanacearum* without displaying wilt symptoms. Nakaho *et al.* (2004) revealed that bacterial multiplication in the stems of resistant tomato plants was suppressed due to limited pathogen movement from the protoxylem or primary xylem to other xylem tissues (85). A proteomic approach was used to elucidate molecular interactions in the cell walls of resistant and sensitive plants inoculated with *R. solanacearum* (23). Resistance to bacterial wilt in many crops has generally been negatively correlated with yield and quality. Thus, the release of resistant cultivars may be poor because of other agronomic traits and are not widely accepted by farmers or consumers. The breeding of a good resistant cultivar is expected in the future through stronger efforts in the genetic enhancement of bacterial wilt resistance through biotechnology approaches in order to improve yield crop.

#### 2) Crop rotation, multi-cropping

The benefits of crop rotation are maintenance of the soil structure and organic matter, and a reduction in soil erosion that is often associated with continuous row crops (51). While continuous cropping with the same susceptible host plant will lead to the establishment of specific plant pathogenic populations, crop rotation avoids this detrimental effect and is often associated with a reduction in plant diseases caused by soil-borne pathogens (51, 66). For example, the onset of bacterial wilt was delayed by 1 or 3 weeks and wilt severity was reduced by 20–26% when a susceptible tomato variety was grown after corn, lady’s fingers, cowpea, or resistant tomato (4). Potato cultivation rotated with wheat, sweet potato, maize, millet, carrots, sorghum, or phaseolus beans reduced the incidence of wilt by 64 to 94% while the yield of potatoes was 1- to 3-fold higher than that of monocultured potatoes (54). In an example of multi-cropping, Yu *et al.* (140) reported the suppression mechanisms of Chinese chive (*Allium tuberosum*), which reduced the incidence of bacterial wilt in the tomato (approximately 60%) because the root exudates of Chinese chive may prevent *R. solanacearum* from infecting tomato plants.

#### 3) Soil amendment

Previous studies revealed that the application of fertilizers reduced the incidence of bacterial wilt. Calcium (Ca) is the most well-known fertilizer to suppress disease. Increased Ca concentrations in plants reduced the severity of bacterial wilt as well as the population of *R. solanacearum* in the stems of the tomato (134, 136). Furthermore, an increase in Ca uptake by tomato shoots correlated with lower levels of disease severity (135, 136). Lemaga *et al.* (68) reported that the application of nitrogen (N) + phosphorus (P) + K and N + P (application rate of each fertilizer = 100 kg ha^−1^) reduced bacterial wilt by 29% and 50%, respectively, and increased the yield of potatoes to 18.8 t ha^−1^ and 16.6 t ha^−1^, respectively, which was higher than that in untreated controls (11.2 t ha^−1^). Hacisalihoglu *et al.* (38) reported that bacterial wilt induced changes in the distribution of nutrients, especially Ca, B, and P in tomato leaves. Li and Dong (70) showed that the combined amendment of rock dust and commercial organic fertilizer reduced the incidence of bacterial wilt in the tomato. A single amendment with rock dust also effectively reduced the incidence of bacterial wilt in the tomato and higher soil pH and Ca content were key factors in the control of bacterial wilt by the rock dust amendment.

Many elements in the cell walls influence the susceptibility or resistance of plants to infections by pathogens and silicon is considered to be a beneficial element for plants and higher animals (29). Kiirika *et al.* (60) reported that the combined application of silicon and chitosan reduced the incidence of bacterial wilt in the tomato by inducing resistance. Si and chitosan exhibited synergistic effects against the disease. *Integrated Pest Management (IPM)*

According to Agrios (5), the main goals of an integrated plant disease control program, regarded as integrated pest management (IPM), are to (i) eliminate or reduce the initial inoculums, (ii) reduce the effectiveness of initial inocula, (iii) increase the resistance of the host, (iv) delay the onset of disease, and (v) slow secondary cycles.

IPM reduced bacterial wilt disease by 20–100% in the field or under laboratory conditions, and typically combines two or three methods among cultural practices and chemical and biological methods. For example, the incidence of bacterial wilt in the tomato was monitored in soil infested with *R. solanacearum* and the addition of an organic mixture consisting of agricultural and industrial waste such as bagasse, rice husks, oyster shell powder, urea, potassium nitrate, calcium superphosphate, and mineral ash or Actigard (active ingredient: acibenzolar-*S*-methyl [ASM]). The addition of the organic mixture decreased the incidence of bacterial wilt in the tomato by 32%, while that of Actigard decreased it by 5%. In contrast, the addition of the organic mixture and Actigard decreased the incidence of bacterial wilt by 53% (12). We previously demonstrated that suppressive effects against bacterial wilt in the tomato were enhanced by combinations of BCAs and their substrates, such as lysine, sucrose, and anaerobically digested slurry, in which the addition of substrates improved the colonization of tomato roots by BCAs (87, 88).

The relative importance of factors accounting for production losses need to be assessed in order to develop IPM. Combinations in cultural practice methods, such as the combination of crop rotation with a resistant cultivar or a soil amendment, or the combination of organic matter with a non-pesticide chemical such as formaldehyde or bleaching powder appear to have effectively reduced the incidence of bacterial wilt and increased crop yield (4, 68, 108, 121). The combined application of ASM and *P. fluorescens* Pf2 resulted in the greatest reduction in the incidence of bacterial wilt in the tomato, while the application of ASM or *P. fluorescens* Pf2 was also effective (1). A previous study reported that the combination of endophytic bacteria (*Bacillus* sp. and *Serratia marcescens*, both of which had no antibiosis) with resistant cultivars of the tomato reduced the incidence of bacterial wilt (18).

Grafting is an important strategy in integrated pest management for soil-borne pathogens. Disease management by grafting has been reported for fungal pathogens (such as *Verticillium*, *Fusarium*, *Pyrenochaeta*, and *Monosporascus*), oomycete pathogens (*Phytophthora*), bacterial pathogens (particularly *Ralstonia*), root knot nematodes, and several soil-borne viruses (78).

We need to select methods that are easy, practical, profitable, and also environmentally healthy to control diseases and improve yields.

### Cautions for disease control measures

#### 1) Keep the environment healthy

Preventive methods are essential for maintaining fields that are free of bacterial wilt. *R. solanacearum* is a soil-borne bacterium and may survive for prolonged periods in soil, water, and plant materials (77). Thus, to keep environment free of this pathogen, it is important to clean seeds, soil, water, and tools in order to improve crop production by preventing this disease. The use of healthy seeds that are free of pathogens is the most economical, environmentally friendly, and effective method for disease control. Cultural practices involving soil amendments, including organic matter, crop rotation, and multi-cropping, can be used to maintain soil health. These agricultural practices influence the chemical, biological, and physical properties of soil, which, in turn, influence the viability and distribution of pathogens as well as the availability of nutrients for pathogens in the soil. Researchers are becoming more interested in investigating the effects of such practices on microbial communities, or in assessing their potential to control soil-borne pathogens. Soil health indicators may be very useful for risk prevision and technical advice (53).

The early detection of *R. solanacearum* in irrigation water or soil is essential for preventing its introduction into new areas. A sensitive quantitative assay was recently developed to detect *Ralstonia solanacearum* in soil by the most probable number (MPN) analysis based on PCR results, in which a pre-culture was performed in a buffer containing antibiotics, but no other carbon source in order to allow the pathogen to grow and to suppress the growth of other soil microorganisms (49). This assay enabled pathogens to be detected at levels as low as 9.3 cfu g^−1^ soil.

#### 2) Abiotic and biotic factors to be considered

Plant diseases caused by soil-borne pathogens such as *R. solanacearum* result from the multiple and complex interactions, including both biotic and abiotic factors, they have with plants. Abiotic factors such as nutrient (organic matter and minerals) conditions, soil type, pH, anaerobic conditions, temperature, and moisture content influence the development of *R. solanacearum* in soil, as described above.

Biotic factors are related to microorganisms, flora, fauna in the soil, and plants that can affect *R. solanacearum*. Previous studies investigated the biotic factors controlling *R. solanacearum* such as the microbial community in soil, introduction of BCAs, cultivar resistance, and rotation, as described above. Various suppression mechanisms are considered to be biotic factors for the pathogen, such as enhanced microbial activity, which can suppress *R. solanacearum*, the release of antibiotics, enhanced competition, decrease in colonization, the induction of systemic resistance, and protection against or avoidance of pathogen contact with the host crop.

#### 3) Economic analysis

Many researchers have managed bacterial wilt with biological, physical, and chemical methods and/or with cultural practices; however, few studies have examined the efficiency of these methods to improve crop yield, especially economic analyses. Based on our reference survey, only 10% of the methods reported improved crop yield. In integrated disease management, soil amendments with 300 kg N and 1,500 kg CaO, together with soil solarization using transparent plastic mulches, reduced the incidence of wilt in the tomato by 20% and increased grower profits, equivalent to 369 to 998 US$ per ha (121). Our primary goal is to contribute to safe, sustainable, and high agricultural production. Attention to cost-benefit analyses is indispensable in the short, middle, and long term.

#### 4) Future of fumigants

The use of a fumigant type of agrochemical in Japan has enabled the establishment of a pathogen-free environment and, thus, intensive agriculture, leading to high quality crop production. However, some of these indiscriminating fumigants are prohibited in European countries, *e.g.* chloropicrin has been banned since 2011 due to the risks posed to pesticide operators and aquatic organisms, birds, and bees (EU No 1381/2011). Due to public concerns and environmental impact, it is not advisable to rely only on single controlling methods, such as fumigants, for high yield and quality crop production. Proper control methods need to be adopted based on the density of and crop resistance to pests.

## Concluding remarks

The research discussed in this review shows how many different diverse options have been reported on control methods against diseases caused by *R. solanacearum*. This unequivocally indicates the importance of these diseases worldwide. The avoidance of crop losses due to pathogens significantly contributes to increased crop production worldwide. We will be able to identify solutions by integrating a biological control agent and organic matter including simple organic compounds, compost, or plant residue.

## Figures and Tables

**Table 1:** Various biocontrol agents that have been tested in the field to control bacterial wilt diseases caused by *Ralstonia solanacearum* (2005–2014)

Microorganisms	Inoculation method and application rate	Mechanisms	BE (%)	Yield*	Ref
1. *Bacillus amyloliquefaciens* SQR-7 and SQR-101 and *B. methylotrophicus* SQR-29	Pouring, 6.8×10^10^ cfu plant^−1^ (SQR-7), 7.5×10^10^ cfu plant^−1^ (SQR-101), 8.2×10^10^ cfu plant^−1^ (SQR-7)	Production of indole acetic acid and siderophores	18–60% in tobacco	25–38%	143
2. *Ralstonia pickettii* QL-A6	Stem injection, 10 μL of 10^7^ CFU mL^−1^	Competition	73% in the tomato	NA	125
3. *Pseudomonas monteilii* (A) + *Glomus fasciculatum* (B)	Stem cuttings were dipped in A (9.1×10^8^ mL^−1^), B (53 infective propagules) was added to each cutting, and A was then poured again	Increased plant nutrient uptake (N, P, K) and reduced the pathogen population	56–75% in herbs (*Coleus forskohli*)	54%	111
4. *Brevibacillus brevis* L-25 + *Streptomyces roche* L-9 + organic fertilizer	Mixed with soil at a density of 7.3×10^7^ (L-25) and 5.0×10^5^ (L-9) cfu g^−1^ of soil	Decreased root colonization by the pathogen	30–95% in tobacco	87–100%	76
5. *Bacillus amyloliquefaciens* + bio-organic fertilizer (BIO23) *B. subtilis* + bio-organic fertilizer (BIO36)	Mixed with soil at a density of 5.5×10^6^ (BIO23) and 7.0×10^6^ (BIO36) cfu g^−1^ of soil	Plant growth promotion	58–66% in the potato	64–65%	26
6. *Bacillus* sp. (RCh6) *Pseudomonas mallei* (RBG4)	3×10^8^ cfu g^−1^ (talc formulation). Seedlings were dipped in antagonist suspension (25 g talc formulation L^−1^). Leftover suspension was poured around the root zone of the seedling (50 mL plant^−1^)	Production of inhibitory compounds and siderophores	81% in the eggplant	60–90%	103
7. *Trichoderma viride* (A), *B. subtilis* (B), *Azotobacter chroococcum* (C), *Glomus fasciculatum* (D), *P. fluorescens* (E)	D (53 infective propagules) was added to each stem cutting that was dipped in A (1.2×10^6^ CFU mL^−1^), B (1.8×10^8^ CFU mL^−1^), C (2.3×10^7^ CFU mL^−1^), and E (2.5×10^8^ CFU mL^−1^). A total of 5 mL of A, B, C, and E was then poured into 200 g soil.	Competition for nutrient uptake (NPK) and reduced *R. solanacearum* population	7–43% in herbs (*Coleus forskohlii*)	159–227%	110
8. *B. amyloliquefaciens* QL-5, QL-18 + organic fertilizer	Mixed with soil at a density of 1×10^7^ (QL-5) or 1×10^7^ (QL-18) cfu g^−1^ of soil	Decreased root colonization by the pathogen	17–87% in the tomato	NA	124
9. *B. amyloliquefaciens* Bg-C31	Poured 10 mL of bacterial suspension plant^−1^ (potato dextrose broth culture).	Production of antimicrobial proteins	60–80% in Capsicum	NA	44
10. *Acinetobacter* sp. Xa6, *Enterobacter* sp. Xy3	Poured 20 mL of the bacterial suspension (1×10^9^ cells mL^−1^) plant^−1^ or seedling roots were soaked in the bacterial suspension.	Rhizocompetence and root colonization	57–67% in the tomato	32–41%	130
11. *B. vallismortis* ExTN-1	Bacterial suspension was mixed into an organic fertilizer (10^6^ cfu mL^−1^) and poured onto soil.	Induction of systemic resistance	48–49% in the tomato	17%	119
12. *Glomus mossease*	A total of 30 g of the inoculum (650–700 spores of *G. mossease* 100 g^−1^ soil) was added to a planting hole.	Competition for nutrients and decreased pathogen population	25% in the tomato	16%	113

BE: biological control efficacy, NA: not applicable, Yield*: increase in yield
